# A Year in Review: Atrial Fibrillation 2025

**DOI:** 10.19102/icrm.2026.17014

**Published:** 2026-01-15

**Authors:** Lydia M. Taranto, Ankur A. Karnik, Rahul N. Doshi

**Affiliations:** 1 Division of Clinical Cardiac Electrophysiology, HonorHealth, Scottsdale, AZ, USA; 2 The John Shufeldt School of Medicine and Medical Engineering at Arizona State University, Phoenix, AZ, USA

**Keywords:** Atrial fibrillation, clinical trials, review, 2025

## Introduction

Welcome back to another year of progress in the management of atrial fibrillation (AF). In the year 2025, we welcomed new technologies, advanced our understanding of existing technologies and pharmaceuticals, continued our movement to perform AF procedures in surgical centers, and began to focus on developing high-performance teams for more comprehensive and standardized care of the AF patient. Let us take a deeper look at some of the hottest AF topics of 2025.

## Ablation technology—pulsed field ablation

International electrophysiology symposia including the Annual Scientific Sessions of the Heart Rhythm Society were filled with presentations of the new and exciting pulsed field ablation (PFA) technology, introducing several new catheters for single-shot ablation (Volt™ [Abbott, Chicago, IL, USA], SPHERE-360™ [Medtronic, Minneapolis, MN, USA]) and a new focal ablation catheter (OMNYPULSE™; J&J Medtech, New Brunswick, NJ, USA). Importantly, as we continue to learn and understand this technology, two of these now present us with the ability to assess tissue contact to hopefully achieve a successful ablation.

### VOLT CE Mark Study

Abbott’s Volt™ catheter is a novel, single-shot, balloon-in-basket catheter with contact force–sensing and three-dimensional (3D) mapping integration.^1^ In the VOLT CE Mark Study, a pre-market single-arm prospective study, 146 patients (age, 64.1 ± 10.0 years; 63.0% men; 70.5% paroxysmal atrial fibrillation [PAF]) with either drug-refractory PAF or persistent AF (PersAF) underwent pulmonary vein (PV) isolation (PVI). At 6 months, the rate of freedom from documented atrial arrhythmias (AAs) was 88.2% in PAF patients and 76.7% in PersAF patients (freedom from symptomatic recurrence was documented in 90.2% of PAF patients and 74.4% of PersAF patients). There were four (2.7%; 4/146) primary serious adverse events: two major vascular access complications, one cardiac tamponade, and one postoperative pneumonia requiring hospitalization. Outcomes in PersAF patients were encouraging, but longer-term data are needed.^1^ This technology has the advantage of being fully integrated into a 3D mapping system that is familiar to most electrophysiologists, facilitating zero or near-zero fluoroscopy as well.

### Omny-IRE

Similarly, the Johnson & Johnson OMNYPULSE™ catheter was presented at the Heart Rhythm Society sessions and is a departure from single-shot PFA catheters.^2^ It is a focal, large-tip, contact force–sensing basket catheter also with full 3D mapping integration.

A Study for the Treatment of Paroxysmal Atrial Fibrillation (PAF) with the OMNYPULSE™ Catheter and the TRUPULSE™ Generator (Omny-IRE) evaluated the safety and effectiveness of PVI in 136 patients with PAF who were evaluated for the primary safety endpoint at 3 months. Prespecified patient subsets underwent systematic brain imaging, esophageal endoscopy, cardiac computed tomography/magnetic resonance angiogram, and mandatory 3-month remapping for PVI durability assessment.

The primary adverse event rate was 3.0% (4/135 patients with 3-month follow-up; 3 with major vascular access complications, 1 with pericarditis). Brain imaging (n = 30) revealed one patient (3.3%) with an asymptomatic silent cerebral event at discharge. No esophageal injury was observed. Computed tomography/magnetic resonance angiography (n = 24) revealed there were no cases of PV narrowing by >70%.

During 3-month remapping, PVI was durable in 62.1% (18/29) of patients. With an optimized workflow, PVI durability improved in 71.4% (15/21) of patients.

These are early data available from a single-arm study, and, especially given this catheter has unique features, we still await data on its clinical efficacy.^2^

### PULSE trial

Medtronic has introduced SPHERE-360™, a second single-shot PFA catheter, to its ablation portfolio. SPHERE-360™ is a large lattice-tip catheter that can conform to the PV ostium, eliminating the need for extensive repositioning.^3^ This was a single-arm study with 1-year follow-up, and three different doses were evaluated.^3^ One hundred PAF patients underwent ablation with PULSE1 (n = 30), PULSE2 (n = 20), or PULSE3 (n = 50) waveforms. After PVI, they were followed up with Holter monitoring at 180 and 365 days and scheduled and symptomatic trans-telephonic monitoring or insertable loop recorder data. Efficacy outcomes were acute PVI and 12-month freedom from AAs, after 90-day blanking. During this trial, the optimal pulsed waveform was not achieved, and so, during the study, invasive remapping at 75 days facilitated proprietary adjustment of the waveform, resulting in waveform refinement from PULSE1 to PULSE2 and subsequently to the optimized PULSE3 approach.

All 395 targeted PVs were acutely isolated, with a transpired PVI time of 11.5 ± 6.0 min, using 4.0 ± 1.3 lesions/PV. There were no primary safety events (serious device-related events within 7 days post-PFA). PVI durability with PULSE3 (n = 40) was 98% (per vein) and 93% (per patient). One-year freedom from AA recurrence was 82.0% (95% confidence interval [CI], 73.0%–88.3%) overall and 88.0% (95% CI, 75.2%–94.4%) for PULSE3 patients specifically.

Interestingly, despite the adjustments made to reach the PULSE3 configuration, the initial PULSE1 and PULSE2 configurations performed well. The opportunity to remap at 75 days after ablating in PULSE1 and PULSE2 showed that only 9% (26 out of 292 veins) of the veins were reconnected, which were then ablated with PULSE3. The study met its primary endpoint of acute vein isolation, and 1-year freedom from AAs was up to 88% with the PULSE3 setting (in a single-arm study).^3^

## Pulsed field ablation: new indications

### ADVANTAGE trial and posterior wall ablation

Since the initial description of focal triggers initiating AF, PVI has remained the only statistically effective ablation strategy for treating AF.^4–6^ Twenty-seven years later, treating PersAF or long-standing persistent AF (LSPAF) remains a challenge, with the data supporting non-PVI sites of ablation being limited. However, posterior wall ablation (PWA) is the most frequently performed non-PVI site in PersAF or LSPAF. A Prospective Single Arm Open Label Study of the FARAPULSE Pulsed Field Ablation System in Subjects with Persistent Atrial Fibrillation (ADVANTAGE-AF) was a prospective, single-arm, multicenter pivotal investigational device exemption study, which looked at the safety and efficacy outcomes of employing PFA to treat PersAF with PWA using the pentaspline PFA catheter (FARAPULSE™; Boston Scientific, Marlborough, MA, USA). A total of 339 patients (260 treatment and 79 roll-in) were enrolled.

The primary safety endpoint was the incidence of predefined adverse events; this was 2.3% (5.1% upper confidence limit), encompassing one patient with pericarditis, one patient with myocardial infarction, and four patients with pulmonary edema. No instance of tamponade, stroke, PV stenosis, or esophageal fistula occurred.

The primary effectiveness endpoints included acute success and post-blanking 1-year freedom from AA recurrence (>30 s), redo ablation, cardioversion, or anti-arrhythmic drug (AAD) escalation. The primary effectiveness was 63.5% (57.3% lower confidence limit) at 1 year, with 8.5% patients having a single, isolated AF recurrence. Freedom from symptomatic AF was 85.3%.^4^

As a result of this work, the U.S. Food and Drug Administration approved an expanded indication of PWA for the pentaspline PFA catheter for refractory PersAF.

### SINGLE SHOT CHAMPION trial

It has barely been 9 years since the FIRE AND ICE trial performed a head-to-head comparison of radiofrequency ablation (RFA) and cryoablation, showing the noninferiority of cryoablation in PAF.^7,8^ Now, PFA has emerged as the new single-shot technology, with perhaps a better safety profile and certainly faster procedural times compared to thermal ablation.^7^ With the increase in the use of PFA, cryoablation use has decreased despite only limited direct comparison, with the FARAPULSE ADVENT PIVOTAL Trial PFA System versus Standard of Care Ablation for Paroxysmal Atrial Fibrillation (ADVENT) demonstrating equivalency.^9^ The SINGLE SHOT CHAMPION trial was performed to look at both technologies head-to-head, with continuous rhythm monitoring. This was a randomized noninferiority trial that evaluated 210 patients with symptomatic PAF, randomly assigned in a 1:1 ratio to undergo PFA (FARAPULSE™) versus cryoablation (Arctic Front; Medtronic). All study participants received an implantable cardiac monitor to detect arrhythmia recurrence. The primary endpoint was the first recurrence of an AA between Days 91 and 365 after ablation. The safety endpoint was a composite of procedure-related complications. Recurrent AA was observed in 39 patients in the PFA group versus 53 patients in the cryoablation group (Kaplan–Meier cumulative incidence, 37.1% and 50.7%, respectively; between-group difference, −13.6 percentage points; 95% CI, −26.9 to −0.3; *P* < .001 for noninferiority, *P* = .046 for superiority). The safety endpoint occurred in one patient (1.0%) with PFA and in two patients (1.9%) with cryoablation.

The results of the SINGLE SHOT CHAMPION trial show that, among patients with symptomatic PAF, PFA was noninferior to cryoballoon ablation with respect to the incidence of a first recurrence of AA.^7^

## Adverse effects of pulsed field ablation

### NEMESIS

Early evidence showed reduced complications with PFA, but non-target tissue collateral damage caused by electroporation effects is poorly understood and may significantly differ between systems.^10^ Initial complications with PFA have been hemolysis of red blood cells (worsened with poor catheter–tissue contact), acute kidney injury due to the hemolysis and release of hemoglobin into the circulation, transient injury to the descending aorta and the phrenic nerve, and coronary vasospasm.^10^

In the NEMESIS study, a multicenter, observational registry of 871 patients who underwent AF ablation with any approved PFA system (either a circular multielectrode array [PulseSelect™; Medtronic], spherical [Affera™ Sphere-9; Medtronic], pentaspline [FARAWAVE™; Boston Scientific], or variable loop catheter [VARIPULSE™; Biosense Webster, Diamond Bar, CA, USA]), the authors evaluated the damaging collateral effects of PFA compared to RFA.

Procedural characteristics, biomarkers for myocardial injury, hemolytic anemia, renal function, and left atrial function in select patients were all assessed.

Significant postprocedural changes in certain biomarkers such as troponin (13,551.0 vs. 127.5 ng/dL; *P* < .001), lactate dehydrogenase (107.5 vs. 26.5 IU/L; *P* < .001), and haptoglobin (−102.0 vs. −33.5 mg/dL; *P* < .001) were detected following the PFA procedures compared to RFA, and the change was dose-dependent. There was a significant change in the left atrial ejection fraction (−20.0% vs. −5.0%; *P* < .001) in PFA versus RFA.

There were significant differences in biomarkers across PFA systems. When stratified by PFA systems, the pentaspline catheter yielded the highest postoperative troponin, lactate dehydrogenase, and plasma-free hemoglobin levels, followed by the spherical catheters, and then RFA.^10^

In this study, it is notable that roughly half of the patients in both groups (PFA and RFA) had PAF, yet all patients received ablations that were “PVI+”—that is, whether PFA or RFA was used, more was done in each ablation than simple PVI. Most participants had posterior wall isolation, and many had additional substrates such as superior vena cava, inferior vena cava, or left atrial appendage (LAA) ablation. One-quarter received an isthmus line ablation, either mitral valve isthmus or cavotricuspid isthmus (CTI). While this is representative of the real world, it also demonstrates that extensive ablation may increase the risk of collateral damage.^10^

### Pulsed field ablation and long-term coronary damage

As with all medical therapies, cardiac ablation has its own risks.^11^ One such risk is long-term coronary artery intimal hyperplasia, if not chronic stenosis, if ablation has been performed within 5 mm of the artery. This has been demonstrated with RFA in an ovine model, where intimal and medial hyperplasia were seen, but not clinically significant stenosis.^12^ Large cohort studies of RFA showed no signal of coronary damage, but this was based only on angiography and not on intracoronary imaging.^11^ During RFA near an artery, the brisk flow of the artery acts as a “heat sink,” mitigating damage to the vessel. PFA is known to cause acute coronary spasm, but whether this translates into long-term coronary artery stenosis is unknown. An elegant study by Tam et al. sought to evaluate changes in coronary arteries after PFA for atrial flutter as evaluated by optical coherence tomography (OCT).^11^

In a study of 21 (with 2 later removed for pre-existing lesions) patients undergoing PVI and additional lines either at the mitral isthmus or CTI with PFA (FARAPULSE™), patients underwent coronary angiography and OCT before and immediately after ablation. Bolus intracoronary nitroglycerine was given before and throughout ablation. Three months after ablation, patients underwent repeat OCT imaging.

Nineteen patients had paired imaging data for 20 coronary vessels (18 right coronary and 2 left circumflex arteries). At 3 months, the vascular wall area at the ablation site increased by a median of 0.40 mm^2^ (Q1–Q3, 0.13–0.71 mm^2^; *P* < .01), or 17.1% (Q1–Q3, 8.6%–31.0%). The median reduction in the luminal area was 0.70 mm^2^ (Q1–Q3, 0.18–1.30 mm^2^; *P* < .01), or 10.1% (Q1–Q3: 4.7%–16.2%). This change did not lead to clinically significant stenosis.

Additionally, it appears that 3-month loss in luminal area may be correlated with the degree of acute spasm (Spearman correlation coefficient, 0.42; *P* = .08). This suggests that spasm potentially reflects the vascular proximity to the energy source and can be a surrogate for the prediction of subsequent coronary stenosis.

Interestingly, despite pre-treatment with nitroglycerin, only 5 of the 20 patients did not experience acute coronary spasm (whether mild, moderate, or severe), suggesting that the majority of patients who receive PFA near a coronary artery will develop some degree of spasm.

Taken together, these data show that, although intracoronary vasodilator therapy may prevent or treat acute spasm, the risk of arterial stenosis remains, calling for heightened vigilance and long-term follow-up.^11^ Interestingly, the decrease in luminal area after PFA resembles the intimal and medial hyperplasia seen in animal models of RFA, but, as our RF data are based only on coronary angiography and not on intracoronary imaging such as OCT,^11^ it is unclear whether these two ablation modalities differ in their long-term impacts on coronary vasculature.^11^

## Autonomic modulation after atrial fibrillation ablation

### CIRCA-DOSE: long-term differences in autonomic alterations after cryoballoon versus radiofrequency atrial fibrillation ablation

It is well established that performing PVI ablation may also affect the autonomic nervous system given the proximity of the venous antrum to the ganglia plexi.^13^ While there is considerable evidence that the autonomic nervous system contributes to the initiation and maintenance of AF, the long-term natural history of ablation-induced autonomic alterations remains poorly defined due to studies lacking long-term monitoring.^13^ In the Cryoballoon versus Irrigated Radiofrequency Catheter Ablation: Double Short versus Standard Exposure Duration (CIRCA-DOSE) study, the authors sought to define the long-term impact of thermal catheter ablation on the cardiac autonomic system.^13^

A total of 346 patients with drug-refractory PAF undergoing PVI using thermal ablation energy (RFA or cryoballoon ablation) underwent insertion of a Reveal LINQ™ implantable cardiac monitor (Medtronic) prior to ablation, which continuously recorded physical activity, heart rate variability (HRV) (measured as the standard deviation of the average normal-to-normal), daytime heart rate, and nighttime heart rate. Longitudinal autonomic data of the 2-month period prior to the date of ablation were compared with the 3-year data following ablation.

Following ablation, there was a significant decrease in HRV (10–20 ms; *P* < .0001) and significant increases in both daytime heart rate (10 bpm; *P* < .0001) and nighttime heart rate (7 bpm; *P* < .0001), respectively. Changes in autonomic parameters were greatest in the first 3 months following ablation but remained significantly different from baseline for 36 months following ablation. Greater changes in nighttime heart rate and HRV were associated with improved freedom from recurrent AA. The type of thermal ablation energy had no influence on the heart rate and autonomic parameters.

Based on these data, PVI with thermal ablation energy results in significant sustained changes in the heart rate parameters related to autonomic function. These changes are independent of the ablation technology employed and are associated with procedural success.^13^

Of note, in our year-end summary of 2024 in the journal, we discussed that PFA may not affect autonomic tone.^14^ However, in a subanalysis from the ADVENT trial, the authors compared HRV following PFA versus thermal ablation. Compared to PFA, thermal ablation led to significantly greater increases in the heart rate from baseline to 6 and 12 months as measured by Holter monitoring (10 vs. 6 bpm). HRV was lower at both 6 and 12 months after thermal ablation. Both parameters are markers of decreased vagal tone, and, by comparison, PFA did not have the same effect on HRV or baseline heart rate.^14^ This was despite the demonstrated equivalent efficacy of PFA when compared to thermal ablation. This suggests that additionally targeting ganglia plexi during ablation for AF may not be of great utility except in specific patient subsets, such as younger patients with vagally mediated AF.^9,14,15^

## Atrial fibrillation mapping technology

### TAILORED-AF trial

While PVI is the cornerstone procedure for all AF ablations, the optimal ablation procedure for PersAF and LSPAF remains elusive.^16^ There has been some interest in targeting spatiotemporal electrogram dispersion in addition to PVI for these patients.^16^ In the Tailored versus Anatomical Ablation Strategy for Persistent Atrial Fibrillation (TAILORED-AF) trial, the deep-learning algorithm Volta AF-Xplorer was used to identify targets of spatiotemporal electrogram dispersion for ablation in PersAF patients (in addition to PVI), resulting in a procedure tailored to the patient.^16^ The trial is a multicenter, randomized controlled, double-blind superiority trial of patients with drug-refractory PersAF. These patients were randomly assigned to either a targeted approach in addition to PVI (tailored arm, *n* = 187, 23% women) or a conventional PVI-only procedure (anatomical arm, *n* = 183, 19% women).

The primary efficacy endpoint was freedom from documented AF with or without AADs at 12 months after a single ablation procedure. Secondary endpoints included freedom from any AA events and a secondary composite safety endpoint of death, cerebrovascular events, or treatment-related serious adverse events.

The trial met its primary efficacy endpoint for superiority, with freedom from AF at 1 year achieved in 88% of patients in the tailored arm compared to 70% of patients in the anatomical arm (log-rank *P* < .0001 for superiority). For the secondary endpoints, there was no significant difference between the two arms for any AA or safety endpoint. It should be noted that the procedure and ablation times were twice as long in the tailored arm compared to PVI alone.^16^

### SUPPRESS-AF trial

While we have excellent data for PVI ablation for PAF, the results of PVI alone in PersAF have been disappointing.^17^ Atrial cardiomyopathy, which develops as a result of AF,^18^ produces low-voltage areas (LVAs) and diseased tissue that are thought to potentially be non-PV triggers of AF in PersAF, although this has not been definitively shown. Therefore, some have considered a strategy of ablation of these LVAs in conjunction with PVI in PersAF patients, but the efficacy of this approach has not previously been determined.^17^

Efficacy and Safety of Left Atrial Low-voltage Area Guided Ablation for Recurrence Prevention Compared to Pulmonary Vein Isolation Alone in Patients with Persistent Atrial Fibrillation (SUPPRESS-AF), a multicenter, randomized controlled trial, investigated the efficacy of LVA ablation in patients with PersAF and left atrial LVAs that covered ≥5 cm^2^ of the left atrial surface on a voltage map, after PVI was complete. A total of 1347 patients (1003 men and 344 women) who underwent initial ablation for AF were evaluated for LVAs. A total of 341 patients with left atrial LVAs were randomized to the PVI + LVA-ABL (n = 170) or the PVI-alone group (n = 171).

Recurrence of AF or atrial tachycardias (ATs) was monitored using 24-h Holter electrocardiography (ECG) and twice-daily portable ECG recordings. The primary endpoint was freedom from AF or AT recurrence without AAD use during 1 year of follow-up. Although the PVI + LVA-ABL group demonstrated a numerically higher rate of freedom from AF or AT recurrence compared to the PVI-alone group (61% vs. 50%), this difference did not reach statistical significance (*P* = .127).^17^ While this was a negative trial (compared to TAILORED-AF), it does reinforce the idea that the optimal ablation strategy for PersAF is still unknown.

## Atrial fibrillation ablation in ambulatory surgery centers

As the demand for AF ablation rises, health care systems have felt the strain of a lack of beds for overnight monitoring postoperatively.^19^ A popular solution has arisen in same-day discharge, and, taking a step further, ablation has been performed in ambulatory surgery centers (ASCs) with same-day discharge. In a study by Kanthasamy et al., the safety and feasibility of CA for AF in an ambulatory day surgery center outside the hospital setting were evaluated.^19^ This was a retrospective study of consecutive patients who underwent CA for AF at a newly established outpatient surgical center between 2020 and 2024. Eligibility criteria included a body mass index of <45 kg/m^2^ and undergoing either first-time PVI or redo PVI/AT ablation. Procedures were performed under general anesthesia with transesophageal echocardiography-guided transseptal puncture. Of note, all operators were deemed experienced cardiac electrophysiologists, each with a minimum of 5 years of experience and performing at least 50 AF ablations annually.

A total of 450 patients underwent AF ablation, with a median age of 61 (54–69) years, and 95% were undergoing their first AF ablation. Cryoballoon ablation was performed in 350 patients (78%), and 80% had PAF. The median procedure duration was 58 (50–70) min, with successful PVI achieved in all cases. Two patients required same-day hospital transfer but were managed conservatively. The overall acute procedural adverse event rate was 1.2%, with no cases of tamponade or major complications requiring intervention. Three patients (0.6%) required medical attention within 30 days after the procedure.

This large single-center experience represents the first report in Europe, demonstrating that AF CA in an ASC is both safe and feasible. Numerous caveats remain, however, including that the majority of ablations in this study were de novo ablations, done with cryoablation (which has now largely been supplanted by PFA), with the support of general anesthesia, in lower-risk patients with PAF, and in the hands of experienced operators.^19^

Recently, the US Centers for Medicare and Medicaid Services (CMS) released the 2026 Hospital Outpatient Prospective Payment System and ASC Payment System proposed rule on July 15, 2025, which included a proposal to add cardiac catheter ablation procedures to the ASC covered procedures list. This has recently received approval.

## COOPERATIVE-PFA

PFA can cause significantly more pain to the patient secondary to skeletal muscle stimulation, which makes it more difficult to perform without general anesthesia.^20^ The Conventional versus Optimised Periprocedural Analgosedation versus Total Intravenous Anaesthesia for Pulsed-Field Ablation (COOPERATIVE-PFA) trial sought to investigate a protocol for deep analgosedation (DAS) that may keep patients comfortable and reduce sedation-related adverse events, facilitating a PFA for AF without the need for general anesthesia. The investigators compared ketamine–remimazolam DAS (remimazolam being an extremely short-acting benzodiazepine and ketamine having a long-established favorable profile for deep sedation) and propofol–opioid total intravenous anesthesia (TIVA) with propofol–opioid DAS, focusing on sedation-related adverse events.

A total of 127 patients with PAF presenting for PFA CA for AF were randomly assigned in a 1:1:1 ratio to (1) DAS using intermittent propofol–opioid boluses (arm P), (2) continuous remimazolam–ketamine DAS (arm R), or (3) continuous propofol–opioid TIVA with secured airway (TIVA arm). All regimens were administered and monitored by both an anesthesiologist and an anesthesia assistant.

It is worth noting that the major exclusion criterion included heart failure with New York Heart Association Functional Classification III or IV, significant valvopathy, or obstructive sleep apnea syndrome (with an apnea–hypopnea index of >30 points).

The primary endpoint was a composite of hypoxemic, hypotensive, or hypertensive events requiring intervention or leading to procedure discontinuation. Secondary endpoints included hemodynamic instability events, procedure time, serious adverse events, and patient satisfaction.

The primary endpoint occurred in 85.7% of patients in the P arm, 27.9% of patients in the R arm, and 66.7% of patients in the TIVA arm (*P* < .001), driven by hypoxemia in the P arm (100% of patients with the primary endpoint) and by hypotension in the TIVA arm (100%). The R arm showed a similar distribution of hypoxemia (50%) and hypotensive (66.7%) events. No differences were observed in mean procedural time, the rate of serious adverse events, or patient satisfaction.

Based on these data, in PFA procedures for PAF performed on patients without significant risk, remimazolam–ketamine DAS was superior to propofol–opioid regimens (either bolus or continuous) and had the lowest risk of hypoxemia and hypotensive events, when administered and monitored by an anesthesiologist. Importantly, patient satisfaction was the same in all three groups.^20^

## Left atrial appendage occlusion

### Three-year OPTION trial follow-up

In our 2024 review, we discussed the Comparison of Anticoagulation with Left Atrial Appendage Closure After AF Ablation (OPTION) trial.^21^ However, in April 2025, the 36-month data were published. OPTION was an international randomized trial involving 1600 patients with AF who had an elevated score (≥2 points in men and ≥3 points in women) on the CHA_2_DS_2_-VASc scale and who underwent catheter ablation (non-PFA, as PFA technology was not available at the time). Patients were randomly assigned in a 1:1 ratio, with 803 patients assigned to undergo LAA closure (followed by 90 days of anticoagulation, then aspirin alone) and 797 assigned to receive oral anticoagulation indefinitely. The mean CHA_2_DS_2_-VASc score was 3.5 ± 1.3 points.^21^

The primary safety endpoint was non–procedure-related major bleeding or clinically relevant nonmajor bleeding at 36 months, evaluated for superiority. This occurred in 65 patients (8.5%) in the LAA closure group (device group) and in 137 patients (18.1%) in the anticoagulation group (*P* < .001 for superiority).

The primary efficacy endpoint was a composite of death from any cause, stroke, or systemic embolism at 36 months, evaluated for inferiority. This occurred in 41 patients (5.3%) in the LAA closure group and 44 patients (5.8%) in the anticoagulation group (*P* < .001 for noninferiority).

In summary, in patients with an average CHA_2_DS_2_-VASc score of 3.5 points, LAA occlusion (LAAO) at the time of ablation was superior with regard to the primary safety endpoint of non–procedure-related bleeding over 3 years and noninferior with regard to the primary efficacy endpoint of the composite of death, stroke, or systemic embolism over 3 years.^21^

## Pharmacotherapy for atrial fibrillation

### ALONE-AF

It has long been held that patients with AF develop an atrial cardiomyopathy that is thrombogenic, regardless of whether the patient is actively in AF or not, and therefore patients with increased stroke risk (CHA_2_DS_2_-VASc score >2 points) remain on anticoagulation indefinitely post-ablation.^18,22^ However, there are no strong randomized controlled trial data to move recommendations from equipoise on this issue.^22^ Any anticoagulation certainly increases a patient’s risk of bleeding (Apixaban Versus Acetylsalicylic Acid [ASA] to Prevent Stroke in Atrial Fibrillation Patients Who Have Failed or Are Unsuitable for Vitamin K Antagonist Treatment [AVERROES] trial).^23^ OPTION has just shown us that, at 36 months, a strategy of LAAO and ASA post-ablation (after 90 days of anticoagulation) is both superior to long-term anticoagulation, in terms of bleeding events, and noninferior for preventing strokes.^21^

Anticoagulation One Year After Ablation of Atrial Fibrillation in Patients with Atrial Fibrillation (ALONE-AF) was a randomized clinical trial that evaluated the outcomes of cessation of anticoagulation in patients who had no recurrence of their AF 1 year post-ablation. A total of 840 adult patients (aged 19–80 years), 67.6% of whom had PAF, were enrolled at 18 hospitals in South Korea. The patients were randomly assigned in a 1:1 ratio to discontinue oral anticoagulants (OACs) (n = 417) or continue OACs (with direct OACs; n = 423). Recurrence of AF was evaluated by short-term external monitors (or guided by symptoms), not by long-term implantable monitoring. Patients had an average age of 60 years, and 70% were men, with 66% having PAF and the average CHA_2_DS_2_-VASc score being 2 points.

The primary outcome was the first occurrence of a composite endpoint of stroke, systemic embolism, and major bleeding at 2 years. Individual components of the primary outcome were assessed as secondary outcomes. The primary outcome occurred in one patient (0.3%) in the “discontinue” OAC group versus eight patients (2.2%) in the “continue” OAC group (absolute difference, −1.9 percentage points; 95% CI, −3.5 to −0.3; *P* = .02). The 2-year cumulative incidence of ischemic stroke was 0.3% in the “discontinued” OAC group versus 0.8% in the “continued” OAC group (absolute difference, −0.5 percentage points; 95% CI, −1.6 to 0.6). Major bleeding occurred in zero patients in the “discontinued” OAC group versus five patients (1.4%) in the “continued” OAC group (absolute difference, −1.4 percentage points; 95% CI, −2.6 to −0.2).

From these results, we can find that, in East Asian patients (predominantly men), with an average CHA_2_DS_2_-VASc score of 2 points and no documented recurrence of AF for 1 year post-AF ablation, discontinuing OAC therapy resulted in a lower risk for the composite outcome of stroke, systemic embolism, and major bleeding versus continuing direct OAC therapy.^22^

### OCEAN

One highly anticipated study was presented recently at the American Heart Association meeting in November 2025; Optimal Anticoagulation for Higher-Risk Patients Post-Catheter Ablation for Atrial Fibrillation (OCEAN) was an international, open-label, randomized, blinded outcome assessment trial that looked at the question of whether successful catheter ablation for AF eliminates the need for long-term OAC (compared to single-agent antiplatelet therapy).^24^ A total of 1284 patients who had undergone successful catheter ablation for AF at least 1 year earlier and had a CHA_2_DS_2_-VASc score of ≥1 points were randomly assigned to receive either aspirin (at a dose of 70–120 mg daily) or rivaroxaban (15 mg) and followed up for 3 years.

The primary outcome was a composite endpoint of stroke, systemic embolism, or new covert embolic stroke (defined by one or more new infarcts measuring ≥15 mm on magnetic resonance imaging) at 3 years. The primary outcome occurred in five patients (0.31 events per 100 patient-years) in the rivaroxaban group and in nine patients (0.66 events per 100 patient-years) in the aspirin group (relative risk, 0.56; 95% CI, 0.19–1.65; absolute risk difference at 3 years, −0.6 percentage points; 95% CI, −1.8 to 0.5; *P* = .28).

As part of evaluating the primary endpoint, magnetic resonance imaging of the head was performed after enrollment and at 3 years. New cerebral infarcts measuring <15 mm occurred in 22 of 568 patients (3.9%) in the rivaroxaban group and in 26 of 590 patients (4.4%) in the aspirin group (relative risk, 0.89; 95% CI, 0.51–1.55). Fatal or major bleeding (the composite primary safety outcome) had occurred in 10 patients (1.6%) with rivaroxaban and in four patients (0.6%) with aspirin (hazard ratio, 2.51; 95% CI, 0.79–7.95) at 3 years.

Despite the numerically higher primary endpoints in the aspirin group, the results did not meet statistical significance, and therefore treatment with rivaroxaban did not result in a significantly lower incidence of a composite of stroke, systemic embolism, or new covert embolic stroke than treatment with aspirin.^24^

Although the study did include higher-risk patients, such as those with a CHA_2_DS_2_-VASc score of >3 points and PersAF, a preponderance of study subjects had PAF (67.2%) and a CHA_2_DS_2_-VASc score of ≤2 points (67.9%). This would suggest that, in the relatively lower-risk patient, switching to aspirin only is an acceptable alternative.^24^

### AZALEA TIMI 71

In recent years, factor XI has emerged as a target for anticoagulants due to increasing evidence that it is essential for thrombosis, but nonessential for hemostasis; therefore, factor XI inhibitors have the potential for better safety profiles among anticoagulant drugs.^25^ In 2024, OCEANIC-AF evaluated an oral version of the factor XI inhibitor asundexian but unfortunately was terminated early, as the drug was associated with high stroke or systemic embolism rates compared to apixaban (there were, as anticipated, fewer major bleeding events with asundexian).^26^

Abelacimab, whose safety was studied in the 2025 Safety and Tolerability of Abelacimab (MAA868) versus Rivaroxaban in Patients with Atrial Fibrillation (AZALEA TIMI 71), is an injectable, fully human monoclonal antibody that binds to the inactive form of factor XI and blocks its activation (whereas asundexian only inhibited factor XIa activity). The primary endpoint of AZALEA TIMI 71 was the evaluation of its safety as compared with a direct OAC in patients with AF.

Patients with AF and a moderate-to-high risk of stroke were randomly assigned, in a 1:1:1 ratio, to receive subcutaneous injection of abelacimab (150 mg or 90 mg once monthly) administered in a blinded fashion or oral rivaroxaban (20 mg once daily) administered in an open-label fashion. The primary endpoint was major or clinically relevant nonmajor bleeding. A total of 1287 patients underwent randomization; the median age was 74 years, and 44% were women.

The incidence rate of major or clinically relevant nonmajor bleeding was 3.2 events per 100 person-years with 150 mg of abelacimab and 2.6 events per 100 person-years with 90 mg of abelacimab, as compared to 8.4 events per 100 person-years with rivaroxaban (hazard ratio for 150 mg of abelacimab vs. rivaroxaban, 0.38 [95% CI, 0.24–0.60]; hazard ratio for 90 mg of abelacimab vs. rivaroxaban, 0.31 [95% CI, 0.19–0.51]; *P* < .001 for both comparisons). The incidence and severity of adverse events appeared to be similar in the three groups.

The trial was stopped early on the recommendation of the independent data monitoring committee because of a greater-than-anticipated reduction in bleeding events with abelacimab. This trial was not designed to evaluate the efficacy of abelacimab in preventing thromboembolic events. Numerically, in this study, strokes were more frequent in the abelacimab group than in the rivaroxaban group, despite the incidence rate of stroke or systemic embolism being low (approximately 1% per year). However, treatment with abelacimab resulted in markedly lower levels of free factor XI and fewer bleeding events than treatment with rivaroxaban.^25^

At this time, the phase 3 Study to Evaluate the Efficacy and Safety of Abelacimab in High-risk Patients with Atrial Fibrillation Who Have Been Deemed Unsuitable for Oral Anticoagulation (LILAC-TIMI 76) is fully underway in evaluating the efficacy of abelacimab in preventing stroke compared to placebo. This study is anticipated to reach its completion in October 2026.

### EPIC-CAD

The Edoxaban Versus Edoxaban with Antiplatelet Agent in Patients with Atrial Fibrillation and Chronic Stable Coronary Artery Disease (EPIC-CAD) trial was a multicenter, open-label, adjudicator-masked, randomized trial comparing edoxaban monotherapy versus dual antithrombotic therapy (edoxaban plus a single antiplatelet agent) in patients with stable coronary artery disease (defined as coronary artery disease previously treated with revascularization or managed medically) and AF (PAF, PersAF, or permanent), with a CHA_2_DS_2_-VASc score of ≥2 points.^27^

The primary outcome was a composite of death from any cause, myocardial infarction, stroke, systemic embolism, unplanned urgent revascularization, and major bleeding or clinically relevant nonmajor bleeding at 12 months. Secondary outcomes included a composite of major ischemic events and the safety outcome of major bleeding or clinically relevant nonmajor bleeding.

Approximately 500 patients were randomized to each group. The average CHA_2_DS_2_-VASc score was 4.3 points; 55% of the patients in each group had PAF, and 45% had PersAF or permanent AF. At 12 months, a primary outcome event had occurred in 6.8% with edoxaban monotherapy and in 16.2% with dual therapy (hazard ratio, 0.44; 95% CI, 0.30–0.65; *P* < .001).

The cumulative incidence of major ischemic events at 12 months appeared to be similar in both trial groups. Major bleeding or clinically relevant nonmajor bleeding occurred in 4.7% in the edoxaban monotherapy group versus 14.2% in the dual antithrombotic therapy group (hazard ratio, 0.34; 95% CI, 0.22–0.53).

Based on these results, in select patients with AF and stable coronary artery disease, edoxaban monotherapy led to a lower risk of a composite of death from any cause, myocardial infarction, stroke, systemic embolism, unplanned urgent revascularization, and major bleeding or clinically relevant nonmajor bleeding at 12 months; this result appeared to be primarily driven by the reduced risk of bleeding. The incidence of ischemic events in both groups remained similar in both groups at 12 months.^27^

## Lifestyle—PRAGUE-25

The Catheter Ablation versus AADs and Risk Factor Modification trial (PRAGUE-25) sought to evaluate the impact of lifestyle modification (LFM, defined as weight loss and physical exercise) in combination with AAD therapy versus catheter ablation in patients with obesity and AF.^28^ A total of 212 patients with a body mass index of 30–40 kg/m^2^ and either PAF or PersAF were randomized to catheter ablation versus LFM + AAD. Ultimately, 203 patients were included in the final analysis; 100 patients were in the catheter ablation group and 103 were in the LFM + AAD group, with a mean follow-up of 23.5 months. Seven-day electrocardiographic Holter recordings were performed every 3 months to evaluate AF burden. The primary endpoint was freedom from AF at 12 months after randomization. Secondary endpoints included AF burden, changes in metabolic parameters, and quality of life as assessed with the Atrial Fibrillation Effect on Quality of Life questionnaire.

The percentage of patients with freedom from AF at 12 months was 73.0% in the catheter ablation group and 34.6% in the LFM + AAD group (*P*_noninferiority_ = .99, *P*_superiority_ < .001). As one might expect, weight change (−6.4 ± 7.9 kg vs. −0.35 ± 4.8 kg; *P* < .001) and decreased hemoglobin A1c were more significant in the LFM/AAD group than in the ablation group.^28^

This study showed that, despite important metabolic improvements associated with LFM, including weight loss, catheter ablation was still superior to LFM even when combined with AADs.^28^

## Establishing a center of excellence for atrial fibrillation

As we can see from only a single “Year in Review” of AF, our understanding of AF and its treatment is rapidly increasing, together with the complexity of AF therapies.^29^ It is no longer a pathology diagnosed and treated by a single practitioner; effective AF treatment is best accomplished by highly functioning AF centers of excellence (CoEs). In 2025, Bunch et al. have proposed clinical, operational, and continuous quality-improvement criteria that individuals and institutions establishing an AF CoE will need in order to generate and sustain meaningful improvements to enhance the care and outcomes of patients with AF.^29^

They established domains of care that they suggest require standardization, including patient identification and access, care pathways, optimization of electrophysiological procedures, outcomes reporting, education, practice variability, accountability of center performance, and considerations for credentialing. They provided great detail in each category for establishing these AF CoEs, with the goal of maximizing outcomes among patients with or at risk of developing AF.^29^

## Conclusion

The year 2025 was certainly no exception in AF dominating the field of electrophysiology. Just as an artist paints a canvas, using dark and light to reveal the final painting, progress in AF takes shape in both the positive and negative results of our collective research.
